# The effects of the COVID-19 virus on mental healthcare for older people in The Netherlands

**DOI:** 10.1017/S1041610220001040

**Published:** 2020-06-03

**Authors:** Debby L. Gerritsen, Richard C. Oude Voshaar

**Affiliations:** 1 Radboud University Medical Center, Radboud Institute for Health Sciences, Radboudumc Alzheimer Centre, Department of Primary and Community Care, Nijmegen, the Netherlands; 2 University Medical Center Groningen, University Center for Psychiatry and Interdisciplinary Center for Psychopathology of Emotion Regulation, Groningen, the Netherlands

## Importance of the COIVD-19 outbreak in the Netherlands

On March 19, the Dutch government decided to put long-term elderly care and inpatient mental health organizations under quarantine. This means they prohibited visitors from entering the institutions and patients from going outside. Furthermore, outpatient facilities, including daycare and treatment facilities, were closed, and in-person ambulant care was suspended. These measures were taken not only for the safety of patients, but also for that of healthcare staff.

From a health perspective, the COVID-19 pandemic disproportionally affects older persons, as the prognosis of a COVID-19 infection dramatically worsens with increasing age (Wang *et al*., 2020). The measurements taken in the Netherlands, that is, social distancing and self-isolation, may pose an additional burden on older persons with mental illnesses and/or multimorbidity. These patients are, by definition, less resilient to external stressors, feel lonelier on average, and more often need personal assistance with (instrumental) activities of daily living.

Long-term elderly care and mental healthcare receive 19.0% and 6.6%, respectively, of the overall budget for the healthcare system (€ 100 billion in 2018) (CBS, 2020). Around 2,500 long-term elderly care facilities (primarily nursing homes) provide multidisciplinary care and treatment for 115,000 (older) people with neurocognitive disorders and/or physical multimorbidity. Around 60% of nursing home patients have dementia (VERENSO, 2020). Increasingly, Dutch nursing homes have units for specific patient groups such as people with young-onset dementia, Korsakov’s disease, or acquired brain injury. Whereas nursing homes are not allowed to admit patients with a primary psychiatric disorder other than dementia, many nursing homes have developed specific units for older patients with mental–physical multimorbidity (Van den Brink *et al*., 2017). Patients with psychiatric disorders are generally cared for by large mental health organizations offering the full spectrum of specialized mental healthcare. In spite of the trend to scale down long-term mental healthcare, around 1 in 6 psychiatric patients, irrespective of their age, received some kind of long-term care in 2013. Furthermore, around 11% of all patients receiving specialized mental healthcare were aged above 65 years (GGZ, 2013). Despite high prevalence rates of comorbid chronic somatic diseases in these patients, mental health organizations are generally not best suited to treat somatically ill patients. Therefore, the COVID-19 pandemic addresses a significant challenge for these organizations.

In both care sectors, home-dwelling people with psychogeriatric and/or mental disorders have become housebound. As a result, the familiar day structure of older people with these disorders living at home has disappeared, and social isolation may occur. For patients with dementia, this has been reported to lead, for instance, to agitation and fear. For patients with substance use disorders, it leads to risk of relapse and taking risks to get substances. For those with affective disorders, anxiety and depression may worsen, and for those with psychosis, unusual social interactions are likely to increase their paranoia. Furthermore, informal caregivers report an additional burden because of challenging behavior.

## Problems faced during the past several months

In the Netherlands, the first patient with COVID-19 was diagnosed on February 27. In the first week of March, most mental health and nursing home organizations took the first measures to prevent the risk of infection for their patients and staff. These measures were rapidly extended, resulting in social distance, that is, no handshakes and keeping 1.5 meters between persons, as well as the use of personal protective equipment when in contact with infected patients. Staff-only meetings and meetings with patients were converted into conference or video calls. Where possible, staff were asked to work from home to minimize staff infection and thus prevent staff shortages in future weeks. As a result, all group sessions and outpatient visits that were not considered urgent needed to be cancelled. Although all visits to very frail patients were prohibited in nursing homes and mental healthcare units, many mental healthcare organizations chose to allow one visitor per day for 1 hour.

Until the first week of April, no COVID-19 registration mechanism was available for nursing homes, although suspected cases among patients and staff were increasingly reported, particularly in two southern provinces in the country. Therefore, a voluntary registration of COVID-19 was set up as of March 25 by temporarily extending two electronic patient file systems covering most of the Dutch nursing homes. The most recently published data (May 19; in Dutch: https://www.verenso.nl/nieuws/update-covid-19-registratie-uit-ysis-en-ons-19-mei-2020) show that around 9474 had (suspected) COVID-19. Of those, 1779 were deceased and 2122 had recovered. Furthermore, in a subsample of these data, 30% of patients with confirmed COVID-19 died and 13% recovered. It was found that more deaths occurred when more typical symptoms were present (coughing, fever, and dyspnea). Also, 6% of the residents with confirmed COVID-19 showed none of these typical symptoms (https://unovumc.nl/wp-content/uploads/2020/03/Factsheet-1-COVID-19-in-verpleeghuizen-dd-03-05-2020-engels-vertaling-10-mei-2020-def.pdf).

There are no specific data available on COVID-19 infections among care professionals in nursing homes; the most recent figures on Dutch care professionals in general were from April 30 and mention 13,884 having been infected and 9 deaths.

The drastic measures taken on March 19 initially did not seem to lead to discussion; it was simply considered the best thing to do. A Dutch Alzheimer Association survey showed that two-thirds of relatives of nursing home patients with dementia supported all measures taken (in Dutch: https://www.alzheimer-nederland.nl/corona#rol1).

In the past weeks, there has been an outburst of COVID-19 in nursing homes, with around a third of locations reporting infected patients and staff. Probable significant contributing factors included a shortage of personal protective equipment and limited availability of COVID-19 tests. Many mental healthcare organizations and nursing homes only tested patients who were coughing and could have been infected. This also holds for care staff in nursing home and home care. Since this led to fear among care staff, and since more tests have become available as of April 6, tests are increasingly used. On April 12, the government determined a new distribution key between hospitals and nursing homes for protective equipment, increasing the number of materials, and tests available for nursing homes.

The impact of social isolation and quarantine differs per situation and person. On the one hand, more outbreaks of challenging behavior have been reported in nursing home patients who are normally able to go outside and have relatively much control over their daily life, for instance, those with Korskakov’s disease or geropsychiatric conditions. On the other hand, not having visitors is sometimes reported to result in a more tranquil atmosphere on the wards, especially on dementia special care units. Besides the many reports about lonely patients, there are also reports of patients receiving more attention than usual and being more socially engaged among themselves. Preliminary results of a survey about challenging behavior among 300 psychologists and elderly care physicians working in nursing homes indeed show reported increases as well as decreases in challenging behavior and also initial decreases followed by an increase. Furthermore, the participants reported more apathy and depressive complaints (in Dutch: https://www.ukonnetwerk.nl/media/1498/probleemgedragcovidenquete-ukon.pdf).

While two-thirds of relatives supported the measures, many suffered as a result of not being able to visit their loved ones. This suffering and the threatened well-being of patients has led to the Dutch Alzheimer Association asking the government to allow one visitor per patient on April 2. This request was not granted, and the call to allow visitors further intensified in Dutch society. On May 6, the Dutch government announced a limited relaxation of the measures in 25 nursing home locations. As of May 11, these locations are allowing visitors according to a local visitation arrangement based on a national guideline made by stakeholders collectively. Implementation of the local arrangements is currently being monitored globally in all 25 locations, 5 locations are monitored in-depth for compliance, barriers, and facilitators, and impact on patients, visitors, and professionals. Data will be combined with data on infections.

## Solutions already being implemented or considered

Although the focus in nursing homes is predominantly on logistical operations limiting infection and physical aspects, the well-being of patients and care staff is an important point of attention. Psychologists, who are commonly employed by Dutch nursing homes and widely available in mental healthcare settings, have been given prominent, but diverse roles. While psychologists generally support patients and nursing staff in managing patient behavior, they are now asked to also support nursing staff in coping with the COVID-19 crisis, personally and in the teams. Increasingly, nursing staff report anger and fear of infection because of too few protective measures. Psychologists have established a “helpline” for nursing staff in many organizations and provide support for team leaders in managing emotions and group dynamics. Despite these additional tasks, psychologists and other therapists are also expected to help with nursing care when too many nurses fall ill. Furthermore, to compensate for expected illnesses in care workers, there have been calls for former care providers to register to temporarily rejoin the work force. There are reports of up to 20,000 former care providers offering to help during the corona crisis.

There is so much information via (social) media that many mental health professionals risk becoming overwhelmed. Initiatives of nursing platforms, academic long-term care networks, and the Dutch Society of Psychiatry focus on helping them get correct information about taking care of themselves (e.g. keeping the days structured, keeping moving and going out in nature, maintaining social contacts) and taking care of each other (complimenting each other, sharing positive moments, being satisfied with small achievements, accepting that their commitment is greater than their influence).

Since mental health organizations took COVID-19 measures in rapid succession, possibilities for treatment were dramatically reduced. In nursing homes, psychosocial treatment has decreased, although for instance physical activities are continued as much as possible. Direct psychological treatment is limited to patients who are able to use videoconferences. Mediative treatment is more common: psychologists coaching care staff in managing challenging behavior. For home-dwelling people with dementia, several care providers provide day treatment at home. In mental healthcare, outpatient care has been reduced to telephone calls and videoconferences with patients. The first experiences using telepsychiatry have yielded positive and negative experiences (see Table 1).

**Table 1.** First experiences with “telepsychiatry in old age”

	Positive experiences	Negative experiences
**Outpatient care**	Many patients appeared to be much more resilient than health professionals expected.	After the initially resilient period, a substantial proportion (~10%–20%) gradually deteriorated.
	The majority (~60%) of older patients were able to communicate online using videoconferences.	Telephone calls and online video conferences may evoke paranoia in patients prone to psychosis.
	Use of daily diaries to monitor symptoms online (which is appreciated by many more patients, especially by having more time and online support).	No possibility for physical check-ups and blood withdrawal necessary for pharmacotherapy (only used in case of emergency). After the first 8 weeks of online treatment, about one-third of older patients stated to be prepared to take the additional of risk contamination by COVID-19 by coming to the hospital for face-to-face treatment.
**Semi-mural care**	Accelerated development of E-health in geriatric psychiatry.	Not all patients (~35%) were sufficiently skilled to follow group sessions online
	Online group sessions for creative therapy, psychomotor therapy, and vocational therapy were highly appreciated by older patients.	Privacy checks at the patients’ homes are not possible (to prevent partners or visitors seeing or hearing fellow patients).
**Inpatient care**	Volunteer-initiatives by medical students to facilitate additional hygienic measures.	Many older patients refused inpatient care, fearing infection with COVID-19.
		Socially isolated from partner, family and friends.
		Higher disease severity among patients in the inpatient ward (due to patients’ and doctors’ delay in opting for admission).

Although it is too early to provide evidence-based comments on the effectiveness of telepsychiatry in geriatric psychiatry, this rapid transition has encouraged training on the job. For example, professionals need to learn how they can strengthen the therapeutic interaction by moving toward or away from the screen, putting more emphasis on their intonation, and creating different atmospheres.

Nursing homes with COVID-19 have taken different logistical measures. In some, patients with COVID-19 are transferred to especially established coronavirus units within the location or to another location of the same care organization. Other organizations isolate a small-scale unit (6–8 patients) when one of their patients has COVID-19, consequently having to put the other patients at increased risk. In some cases, patients are not allowed to leave their rooms.

While doctors often focus on maximum treatment and cure, many frail older persons prefer less aggressive treatment. Considering the burden of intensive care treatment, the decision whether to opt for critical care should not be obvious or taken lightheartedly. Therefore, we strongly advocate advanced care planning for the most vulnerable patients and carefully listening to the wishes of themselves and their family in case of a complicated course after a COVID-19 infection (see also the paper by Lapid *et al*., in this issue). While this is common practice in Dutch nursing homes, advanced care planning is rarely applied in geriatric mental healthcare.

## Outlook and suggestions for the future

Although there are many local initiatives, the Dutch government has not yet set up a healthcare approach. Based on the experiences in New York City in the aftermath of 9/11 in 2001, this may be relevant to fostering collaboration between agencies in different sectors (long-term elderly care, mental healthcare, social care, local government, etc.) as well as consistent media messages and education (Sederer *et al*., 2011). A national healthcare approach is needed for future care planning as the pressure on the healthcare system will continue for many months. After the current first wave, a second wave is expected regarding acute conditions that are currently not being reported, but may be significant. For instance, the number of newly presented heart conditions currently being reported has decreased (Argulian, 2020). Data from Dutch general practices showed a rapid drop in contacts for non-respiratory, non-COVID-19 health problems, which appeared to be patient-initiated (Schers *et al*., 2020). Thus, it is crucial to avoid a single-minded focus from the health system on the pandemic. Furthermore, a third wave of interrupted care for chronic conditions has been suggested (https://twitter.com/VectorSting/status/1244671755781898241), which is highly relevant for mental healthcare. For instance, demand for primary care for chronic health problems, mental health and prevention decreased when COVID-19 entered the country (Schers *et al*., 2020). Furthermore, there will be delayed consequences of the pandemic, called a fourth wave, such as trauma, burnout, mental illness, and economic problems. It is, therefore, important to address possible physical and mental consequences for care professionals as soon as possible. They will need time to recuperate and many will experience physical and mental complaints in the long term.

To effectively tap into this long-term process, we need staying power. Sometimes, it seems self-evident to fully focus on the needs of our patients. Nonetheless, the saying “You only can take care of other people, if you take care of yourself” might be more relevant than ever during this pandemic.
